# Associations between Social Adversity and Biomarkers of Inflammation, Stress, and Aging in Children

**DOI:** 10.1038/s41390-023-02992-6

**Published:** 2024-01-17

**Authors:** Matthew S. Pantell, Patricia P. Silveira, Euclides José de Mendonça Filho, Holly Wing, Erika M. Brown, Victoria F. Keeton, Irina Pokhvisneva, Kieran J. O’Donnell, John Neuhaus, Danielle Hessler, Michael J. Meaney, Nancy E. Adler, Laura M. Gottlieb

**Affiliations:** 1 Division of Pediatric Hospital Medicine, Department of Pediatrics, University of California, San Francisco, CA USA; 2 Center for Health and Community, University of California, San Francisco, San Francisco, CA USA; 3 Social Interventions Research and Evaluation Network, University of California, San Francisco, CA USA; 4 Douglas Mental Health University Institute, Douglas Research Center, McGill University, Montreal, QC Canada; 5 Ludmer Centre for Neuroinformatics and Mental Health and Department of Psychiatry, Faculty of Medicine, McGill University, Montreal, QC Canada; 6 California Policy Lab, Berkeley, CA USA; 7 Department of Obstetrics, Gynecology, and Reproductive Sciences, University of California, San Francisco, 490 Illinois St, Box 2930, 94143 San Francisco, CA USA; 8 Yale Child Study Center & Department of Obstetrics, Gynecology & Reproductive Sciences, Yale School of Medicine, New Haven, CT USA; 9 Department of Epidemiology and Biostatistics, University of California, San Francisco, CA USA; 10 Department of Family and Community Medicine, University of California, San Francisco, CA USA; 11 Singapore Institute for Clinical Sciences, Agency for Science, Technology and Research (A*STAR), Singapore, Republic of Singapore

## Abstract

**Background:**

Prior work has found relationships between childhood social adversity and biomarkers of stress, but knowledge gaps remain. To help address these gaps, we explored associations between social adversity and biomarkers of inflammation (interleukin-1β [IL-1β], IL-6, IL-8, tumor necrosis factor-alpha [TNF-α], and salivary cytokine hierarchical “clusters” based on the three interleukins), neuroendocrine function (cortisol, cortisone, dehydroepiandrosterone, testosterone, and progesterone), neuromodulation (*N*-arachidonoylethanolamine, stearoylethanolamine, oleoylethanolamide, and palmitoylethanolamide), and epigenetic aging (Pediatric-Buccal-Epigenetic clock).

**Methods:**

We collected biomarker samples of children ages 0–17 recruited from an acute care pediatrics clinic and examined their associations with caregiver-endorsed education, income, social risk factors, and cumulative adversity. We calculated regression-adjusted means for each biomarker and compared associations with social factors using Wald tests. We used logistic regression to predict being in the highest cytokine cluster based on social predictors.

**Results:**

Our final sample included 537 children but varied based on each biomarker. Cumulative social adversity was significantly associated with having higher levels of all inflammatory markers and with cortisol, displaying a U-shaped distribution. There were no significant relationships between cumulative social adversity and cortisone, neuromodulation biomarkers or epigenetic aging.

**Conclusion:**

Our findings support prior work suggesting that social stress exposures contribute to increased inflammation in children.

**Impact:**

Our study is one of the largest studies examining associations between childhood social adversity and biomarkers of inflammation, neuroendocrine function, neuromodulation, and epigenetic aging. It is one of the largest studies to link childhood social adversity to biomarkers of inflammation, and the first of which we are aware to link cumulative social adversity to cytokine clusters. It is also one of the largest studies to examine associations between steroids and epigenetic aging among children, and one of the only studies of which we are aware to examine associations between social adversity and endocannabinoids among children. Clinical Trial Registration: NCT02746393

## Introduction

Social adversity in children is associated with a variety of health outcomes, including poor overall health, poor dental health, and social and emotional problems.^1–3^ Children often experience multiple social risk factors—such as low family income and adverse childhood experiences––and cumulative social adversity experienced during childhood is associated with worse health outcomes both during childhood and adulthood.^2,4–6^ Although social risk factors are related to child health, few studies examine biological mechanisms linking social adversity exposures and health, in part because physical manifestations of disease can be late sequelae. However, recent progress in biomarker development has led to new opportunities to understand the biological pathways through which social adversity contributes to disease states. Understanding variation in biomarker responses based on exposure to social adversity may help to develop more targeted interventions or predict treatment outcomes. For example, prior work has shown that elevated levels of inflammatory markers predict poor response to pharmacologic and behavioral treatments in adults.^7,8^ Based on the promise of biomarkers, in 2021 the American Academy of Pediatrics updated its policy statement on trauma-informed care – practicing care by recognizing and responding to the impact of trauma––noting an “urgent need” for a battery of biological, behavioral, and contextual markers that can stratify risks and predict intervention responses.^9,10^

Social adversity as measured by biological stress activation can be measured through multiple markers, including markers of inflammation, neuroendocrine function, neuromodulation, and epigenetic processes.^11^ Since stress activation influences multiple systems, many authors have noted that a compilation of measures is likely more informative than any single measure. However, outstanding knowledge gaps about the pathways to health and both normative and predictive biomarker values have limited their applications. In this study, we explored associations between social adversity and biomarkers of inflammation, neuroendocrine function (steroid hormone production and metabolism), neuromodulation (endocannabinoids), and epigenetic aging. We hypothesized that children experiencing more social adversity would have higher levels of inflammatory biomarkers, lower levels of endocannabinoid biomarkers, and accelerated epigenetic aging when compared to those experiencing less social adversity.

## Methods

### Sample and recruitment

We performed a sub-analysis of baseline data of a parallel assignment randomized clinical trial (NCT02746393) assessing the effects of a family social needs navigation intervention on pediatric health outcomes.^12^ Details describing study procedures have been described elsewhere.^12^ Briefly, we recruited caregivers of pediatric patients seen in an urban safety-net acute care pediatrics clinic in Northern California from July 2016 through March 2019. Caregiver-child dyads were eligible to participate if they lived in the county of enrollment, the caregiver was familiar with the child’s household environment, the caregiver spoke Spanish or English, and the child was between 0–17 years old. Families were excluded if the child was in foster care, the child was being seen for concern for physical abuse, or if the dyad had enrolled in a similar social needs intervention program 6 months prior to or after recruitment.

Trained research assistants approached families at the clinic on weekdays between 8:30 AM to 5:00 PM to ask if adult caregivers were interested in participating in the study. Among those who were interested in participating, we obtained written informed consent from adult caregivers and verbal assent from children if they were over 7 years old. Research assistants administered most parts of the baseline survey on tablets using Research Electronic Data Capture (REDCap). Caregivers filled out one part of the survey on their own, although they could ask for assistance from the research assistant. The baseline survey was available in both Spanish and English and included questions about patient and caregiver sociodemographic characteristics, family social risks, and families’ priorities about obtaining assistance with social needs. After the baseline survey and biomarker collection were completed, families were randomized into one of two intervention arms, which we will exclude from discussing since we only present baseline data. Families received a $50 gift card after completing the baseline survey and allowing for a child biomarker sample collection, which included a buccal saliva swab and a hair sample. This study was approved by the University of California, San Francisco Institutional Review Board.

### Survey Measures

Caregivers completed the study’s baseline survey about measures of child health and development as well as family sociodemographic factors, including the highest caregiver education level and family income. The survey also asked questions about either the household or child’s social risks, including topics related to food insecurity, unstable housing, utility insecurity, difficulty finding a job, difficulty with unemployment insurance, income support program denial, lack of health insurance, problems with medical/pharmacy bills, lack of access to afterschool activities, lack of access to childcare, bullying, adults in the household with mental or behavioral health issues, transportation needs, and legal problems.

### Biomarker Sample Collection, Storage, and Processing

Given prior work showing associations between some biomarkers of inflammation and childhood adversity, we collected baseline samples from the children of the following inflammatory markers via saliva: interleukin-1β (IL-1β), IL-6, IL-8, and tumor necrosis factor-alpha (TNF-α).^13,14^ We collected hair samples to quantify the following endocannabinoids, neurotransmitters that act on cannabinoid receptors in response to internal and environmental insults and for which evolving evidence suggests a regulatory role in the stress response: *N*-arachidonoylethanolamine (AEA), stearoylethanolamine (SEA), oleoylethanolamide (OEA), and palmitoylethanolamide (PEA).^15,16^ We also collected hair samples to assess steroid hormones (cortisol, cortisone [an inactive metabolite of cortisol], testosterone, and progesterone) and their precursor (dehydroepiandrosterone [DHEA]), as steroids and their precursors have been found to have associations with stress.^17–24^ Finally, using genomic DNA derived from cheek swab samples, we described genome-wide DNA methylation and calculated the Pediatric-Buccal-Epigenetic (PedBE) clock of each child. Epigenetic age as measured by DNA methylation at specific Cytosine-phosphate-Guanine (CpG) sites across the genome is another potential indicator of chronic stress exposure.^25–27^ Epigenetic age acceleration, increased epigenetic age relative to chronological age, is linked to worse health outcomes in adults.^28^ The PedBE clock is a biological marker of aging based on methylation of 94 specific CpG sites. PedBE-derived measures of epigenetic age acceleration have been found to be associated with measures of prenatal adversity.^26^

We collected oral saliva swabs using SalivaBio ORAL Swabs for children over 6 months and using SalivaBio Infant’s Swabs for infants younger than 6 months old (Salimetrics, Carlsbad, United States). The saliva samples were stored at −20 °C before being shipped to the Douglas Mental Health University Institute (Montreal, Canada), where saliva was aliquoted and then sent to the Salimetrics Lab and Technology Center (Carlsbad, United States) for assay. The Salimetrics Lab and Technology Center used a proprietary enzyme-linked immunosorbent assay (ELISA) method that they developed to measure cytokines, measuring them in duplicate using a multiplex assay. We then averaged the two data points per sample.

Steroid hormones and DHEA derived from hair samples were analyzed using a commercial service––hair samples were shipped to the Biochemical Laboratory at TU Dresden. Briefly, hair samples were weighed and washed in isopropranol for 3 min at room temperature then quantified using high-performance liquid chromatography-mass spectrometry. We excluded samples with non-detectable levels of endocannabinoids or steroids.

Cheekswabs were collected from participants using the iSWAB-DNA-250 collection kit (Mawi DNA Technologies). Genomic DNA from cheek swab samples was isolated using an automated approach (Qiasymphony, Qiagen) with DNA concentration and purity assessed using a spectrophotometer. Genome-wide DNA methylation was described using the Infinium MethylationEPIC array according to manufacturer’s guidelines. Signal extraction and DNA methylation data pre-processing (Preprocessnoob function) were performed using the Minfi package in R. We excluded samples with genotype concordance < 0.90 and signal intensity outliers based on control probes. Beta mixture quantile normalization of DNA methylation data was performed using the ChAMP package. Buccal cell proportions were derived using a cell-type deconvolution approach and samples with less than 50% buccal cell content excluded from further analyses.^29^ We calculated estimates of epigenetic age using the PedBE clock as described by McEwen et al., 2020.^30^

Inflammatory markers, steroids, and endocannabinoids were all measured in picograms (pg) per milligram (mg). PedBE clock was measured as a ratio of epigenetic (biological) age to chronological age.

### Data analysis

We log-transformed values of inflammatory markers, endocannabinoids, and DHEA for regression analysis due to their skewed distributions. We transformed testosterone and progesterone using the inverse of their square root values due to the skewness of their log distribution. We additionally created salivary cytokine “clusters” using hierarchical cluster analysis and Ward’s agglomeration method and based on participant cytokine data (IL-1β, IL-6, and IL-8), log-transformed and adjusted for gender and age at collection. This allowed us to categorize participants into one of three clusters based on their aggregate cytokine levels (low, middle, high), with the high cluster representing overall higher cytokine values and a higher burden of inflammation. For cluster creation, we also excluded cytokine values with a coefficient of variation higher or equal to 15% to be consistent with previously described methods.^31^

For PedBE clock analysis, we performed a linear regression predicting epigenetic age based on child chronological age in years and then subtracted the child age in years from the residual predicted from the regression. The resultant value represented how many years older (if the value is positive) or younger (if the value is negative) epigenetic age is compared to chronological age. We excluded values of biomarkers that fell outside three standard deviations of the transformed variables.

We examined associations between each biomarker and the following social factors, which we categorized into approximate quartiles for analysis: highest caregiver education (less than high school, some high school, high school, more than high school), income of the sample ($0–$5 K per year, $5001-$15 K per year, $15,001-$25 K per year, and $25,001 or more per year), and sum of the aforementioned social risk factors collected in the baseline survey as described above (0–1 social risk, 2–3 social risks, 4–6 social risks, 7 or more social risks). Additionally, we constructed a cumulative social adversity variable that assigned one point each for the following: caregiver education less than high school, lowest income group, highest social risk group.

We excluded participants with no biomarker data and missing age or gender data. We calculated regression-adjusted means adjusting for age and gender for each biomarker and compared values by education, income, social risks, and cumulative social adversity using Wald tests. We collapsed participants with a score of 2 or 3 on the cumulative social adversity score due small sample size. For analyses involving PedBE clock, we also adjusted for relevant technical factors (i.e., batch number). We used logistic regression to predict being in the highest cytokine cluster based on the same social predictors while adjusting for patient age and gender. To account for the interaction between biomarkers and illnesses that cause an inflammatory response, we performed a sensitivity analysis of the above Wald tests and logistic regressions additionally adding a dichotomous variable coding for diagnoses related to infection or inflammation as derived from the acute care visit ICD codes. Supplementary Table 1 lists patient ICD codes and whether they were coded as infectious, inflammatory, or neither. As an additional sensitivity analysis, we included a child race and ethnicity variable to the initial Wald tests and logistic regression models (Black non-Hispanic, Hispanic, other race non-Hispanic, and White non-Hispanic).

Significance was set at a *P*-value of less than 0.05. We also conducted significance tests using the Bonferroni correction method, although we discuss results based on a *P*-value of 0.05 in the Discussion section. Analyses were performed from July 2021 to December 2022 using Stata version 17.0 (Stata Corp, College Station, TX).

## Results

**Fig. 1:**
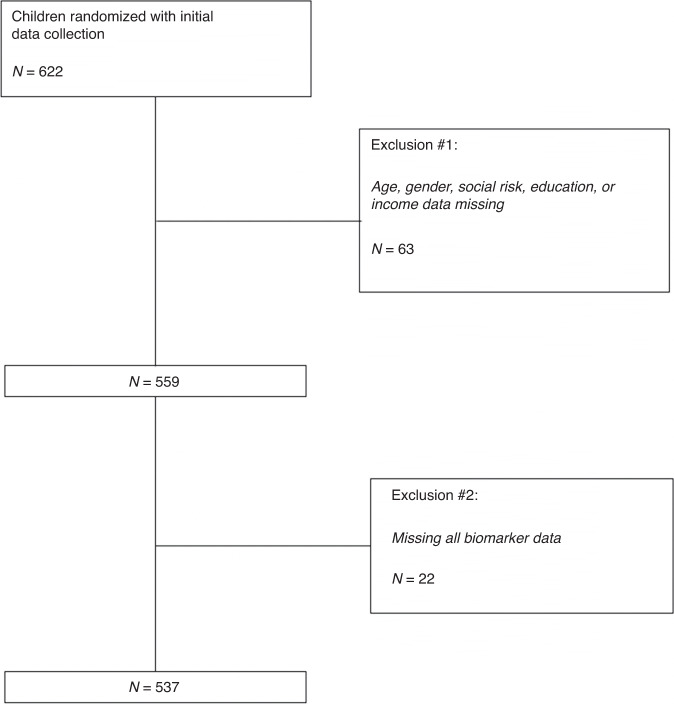
CONSORT Diagram of Participants Included in Study.

Of 622 dyads recruited for the study, our final analytic sample included data from 537 participants because of either missing data or because the caregiver was not the child’s mother, father, or step-parent (Fig. 1). The average age of child participants was 6.3 years, and the majority of children were female, Hispanic, and in either excellent or very good health (Table 1). Most caregivers were between the ages of 25–44 and spoke Spanish (Table 1). Just over half of the sample’s caregivers had a high school degree or more. The average number of social risks per family was 4.3. 47.7% of the sample had a cumulative social adversity score of 0, 37.4% had a score of 1, and 14.9% had a score of 2–3 (Table 1).

**Table 1:** Characteristics of the Sample.

%, unless otherwise noted, *N* = 537	
Child age, mean (standard deviation)	6.3 (5.0)
Child gender	
Female	52.9
Male	47.1
Child race/ethnicity	
Hispanic	78.0
Non-Hispanic Black	9.5
Non-Hispanic White	3.0
Other race/ethnicity	9.5
Baseline health status (*N* = 536)	
Fair/poor	10.8
Good	39.6
Very good	28.5
Excellent	21.1
Caregiver age (*N* = 533)	
18–24	10.5
25–34	38.5
35–44	39.4
45 and older	11.6
Caregiver gender	
Female	89.6
Male	10.4
Caregiver race/ethnicity	
Hispanic	80.8
Non-Hispanic Black	9.7
Non-Hispanic White	3.2
Other race/ethnicity	6.3
Caregiver language	
Spanish	70.2
English	29.8
Caregiver relationship to child	
Mother or father	97.0
Other	3.0
Caregiver education	
Less than 8th grade	24.8
Some high school	24.2
High school graduate or GED	27.9
Some college/college graduate/more	23.1
Income quartile	
Lowest	22.5
2nd	24.4
3rd	23.7
Highest	29.4
No. of social risks, mean (standard deviation)	4.3 (3.2)
Cumulative social adversity score	
0	47.7
1	37.4
2–3	14.9

*N* = 537 unless noted otherwise.

### Associations between biomarkers and individual social factors

We excluded our analysis of testosterone due to a small sample size of eligible samples (*N* = 73).

Regression-adjusted means revealed associations between parent education and child biomarkers of inflammation: increased parental education was associated with decreased levels of inflammatory markers. While these associations were not significant between parent education and both IL-8 and TNF-α, they were significant between parent education and both IL-1β (*P* = 0.043) and IL-6 (*P* = 0.017). Parent education was also significantly associated with AEA (*P* < 0.001), although the association was non-linear; those with some high school education had the highest levels of AEA. Parent education was also significantly related to OEA; those with more education had lower levels of OEA (*P* = 0.039). Finally, parent education was associated with PedBE clock (*P* < 0.001). Similar to the relationship between parent education and AEA, the relationship between PedBE clock and education was non-linear: those with parents with some high school education had the lowest epigenetic age compared to chronological age. There were no other significant associations between biomarkers and parental education (Table 2). There were no significant associations between social variables and inflammatory cluster (Table 3).

**Table 2:** Relationships between Social Adversity and Child Biomarkers.

	IL-1β				IL-6				IL-8				TNF-alpha			
Social Variable	*N* = 484				*N* = 479				*N* = 484				*N* = 480			
**Education**	**RAM and 95% CI**			***P*-value**	**RAM and 95% CI**			***P*-value**	**RAM and 95% CI**			***P*-value**	**RAM and 95% CI**			***P-*value**
<High School	3.672	3.4709	3.87289	0.043*	1.313	1.112	1.51443	0.017*	6.166	5.9888	6.34291	0.178	0.301	0.1518	0.45023	0.202
Some HS	3.449	3.2407	3.65778		1.052	0.8448	1.25925		6.057	5.873	6.24046		0.301	0.1455	0.45667	
High School	3.324	3.1323	3.51615		0.919	0.7283	1.10928		5.974	5.8046	6.14274		0.25	0.1061	0.39311	
>High School	3.299	3.0852	3.51258		0.905	0.6939	1.11658		5.889	5.7005	6.07702		0.091	–0.0682	0.2505	
**Income**	**RAM and 95% CI**			***P*-value**	**RAM and 95% CI**			***P*-value**	**RAM and 95% CI**			***P*-value**	**RAM and 95% CI**			***P*-value**
$0–$5K	3.476	3.2558	3.69638	0.384	1.121	0.9011	1.34031	0.598	6.098	5.9047	6.29175	0.460	0.252	0.0876	0.41645	0.197
$5001–$15K	3.476	3.2771	3.67531		0.961	0.7628	1.15945		6.034	5.8586	6.20843		0.247	0.1	0.394	
$15,001-$25K	3.527	3.3146	3.73917		1.125	0.9132	1.33636		6.077	5.8901	6.26305		0.357	0.2	0.5148	
$25,001 or more	3.297	3.1066	3.48754		1.004	0.8137	1.19472		5.913	5.746	6.08072		0.126	–0.015	0.26732	
**Social Risks**	**RAM and 95% CI**			***P*-value**	**RAM and 95% CI**			***P*-value**	**RAM and 95% CI**			***P*-value**	**RAM and 95% CI**			***P*-value**
0–1	3.538	3.3096	3.76549	0.028*	0.977	0.748	1.20516	0.401	6.013	5.8118	6.21461	0.347	0.22	0.0498	0.3904	0.226
2–3	3.229	3.0425	3.41568		1.064	0.877	1.2519		5.934	5.7688	6.09844		0.175	0.0348	0.31424	
4–6	3.421	3.2323	3.61025		0.962	0.7715	1.15157		6.013	5.8464	6.18031		0.206	0.0652	0.34612	
7 or more	3.648	3.4292	3.86593		1.195	0.9773	1.41298		6.167	5.9738	6.35956		0.389	0.2259	0.55208	
**Cumulative Social Adversity**	**RAM and 95% CI**			***P-*value**	**RAM and 95% CI**			***P*-value**	**RAM and 95% CI**			***P*-value**	**RAM and 95% CI**			***P*-value**
0	3.340	3.1933	3.48603	<0.001**	0.956	0.8102	1.10196	0.001*	5.964	5.8348	6.09254	0.002*	0.227	0.1175	0.33547	0.006*
1	3.375	3.2103	3.53879		0.982	0.8178	1.14558		5.95	5.8058	6.09509		0.144	0.0205	0.26686	
2–3	3.911	3.6478	4.17334		1.505	1.2429	1.76777		6.402	6.1708	6.63351		0.521	0.3254	0.7158	

Cortisol				Cortisone				Progesterone				DHEA				AEA			
*N* = 406				*N* = 409				*N* = 245				*N* = 320				*N* = 400			
**RAM and 95% CI**			***P*-value**	**RAM and 95% CI**			***P*-value**	**RAM and 95% CI**			***P*-value**	**RAM and 95% CI**			***P*-value**	**RAM and 95% CI**			***P*-value**
3.507	3.149	3.86568	0.438	3.325	3.1801	3.46893	0.189	0.692	0.627	0.75742	0.327	1.977	1.7113	2.24277	0.301	–0.121	–0.2006	–0.0405	<0.001**
3.918	3.557	4.27827		3.507	3.3614	3.65256		0.681	0.6217	0.73952		1.89	1.6424	2.13799		0.108	0.0274	0.1887	
3.677	3.3357	4.01847		3.303	3.168	3.43854		0.628	0.5655	0.6908		2.044	1.8032	2.28516		–0.067	–0.1434	0.0091	
3.617	3.2401	3.99379		3.36	3.2106	3.50998		0.708	0.6421	0.77455		1.703	1.4279	1.97761		–0.05	–0.1355	0.03491	
RAM and 95% CI			*P*-value	RAM and 95% CI			*P*-value	RAM and 95% CI			*P*-value	RAM and 95% CI			*P*-value	RAM and 95% CI			*P*-value
3.631	3.2175	4.045	0.989	3.321	3.1575	3.4852	0.401	0.668	0.5983	0.73844	0.974	2.144	1.8529	2.43604	0.155	0.058	–0.035	0.1508	0.070
3.706	3.3532	4.05874		3.35	3.2099	3.49034		0.67	0.6119	0.72825		2.012	1.7634	2.2613		–0.007	–0.0867	0.07202	
3.715	3.3546	4.0747		3.316	3.1704	3.46228		0.679	0.6118	0.74684		1.802	1.5463	2.05806		–0.06	–0.1426	0.02224	
3.66	3.3317	3.9884		3.465	3.3334	3.59594		0.687	0.6266	0.7469		1.767	1.5311	2.00253		–0.093	–0.1676	-0.0189	
RAM and 95% CI			*P*-value	RAM and 95% CI			*P*-value	RAM and 95% CI			*P*-value	RAM and 95% CI			*P*-value	RAM and 95% CI			*P*-value
3.577	3.1742	3.98011	0.411	3.391	3.2303	3.55223	0.228	0.715	0.6464	0.78376	0.150	1.754	1.4576	2.05013	0.511	–0.067	–0.1576	0.02346	0.219
3.719	3.3946	4.04355		3.439	3.3074	3.57133		0.683	0.6239	0.74257		1.952	1.7197	2.18498		–0.026	–0.101	0.04807	
3.88	3.5401	4.21921		3.392	3.2575	3.52654		0.622	0.5633	0.68002		2.024	1.787	2.26011		–0.074	–0.1507	0.00288	
3.465	3.0837	3.84547		3.235	3.0833	3.3862		0.703	0.6361	0.76967		1.845	1.5693	2.12127		0.041	–0.0468	0.12822	
RAM and 95% CI			*P*-value	RAM and 95% CI			*P*-value	RAM and 95% CI			*P*-value	RAM and 95% CI			*P*-value	RAM and 95% CI			*P*-value
3.726	3.4728	3.97864	0.045*	3.412	3.3097	3.51348	0.062	0.679	0.6345	0.72379	0.129	1.84	1.6593	2.02153	0.475	–0.049	–0.1077	0.00881	0.665
3.828	3.5317	4.12349		3.396	3.2781	3.51411		0.645	0.5932	0.69771		2.012	1.8021	2.22251		–0.027	–0.0944	0.04097	
3.134	2.6593	3.60862		3.158	2.9668	3.34934		0.746	0.6629	0.82881		1.912	1.5502	2.27351		0.005	–0.1039	0.11442	

1-Ag				SEA				OEA				PEA				PedBE			
*N* = 403				*N* = 401				*N* = 410				*N* = 411				*N* = 449			
**RAM and 95% CI**			***P*-value**	**RAM and 95% CI**			***P*-value**	**RAM and 95% CI**			***P*-value**	**RAM and 95% CI**			***P*-value**	**RAM and 95% CI**			***P*-value**
4.11	3.9978	4.22173	0.282	6.877	6.723	7.03132	0.081	7.861	7.6786	8.04363	0.039*	7.903	7.7466	8.0602	0.100	0.066	–0.1067	0.2393	0.001*
4.208	4.0943	4.32073		6.705	6.5473	6.86199		7.7	7.5158	7.88342		7.729	7.5723	7.88639		–0.288	–0.4609	-0.1158	
4.118	4.0117	4.22489		6.671	6.5237	6.81861		7.572	7.4003	7.74348		7.742	7.5944	7.88921		0.071	–0.0987	0.241	
4.048	3.9312	4.16436		6.593	6.4304	6.75645		7.505	7.3137	7.69549		7.617	7.4531	7.78116		0.182	0.001	0.3636	
RAM and 95% CI			*P*-value	RAM and 95% CI			*P*-value	RAM and 95% CI			*P*-value	RAM and 95% CI			*P*-value	RAM and 95% CI			*P*-value
4.132	4.0032	4.26085	0.908	6.486	6.3083	6.66377	0.013*	7.477	7.2695	7.68514	0.093	7.685	7.506	7.86351	0.332	0.065	–0.1203	0.24937	0.643
4.15	4.0413	4.25896		6.745	6.5934	6.89576		7.694	7.5157	7.87175		7.756	7.6038	7.90848		–0.093	–0.2697	0.08391	
4.112	3.9993	4.22549		6.878	6.7237	7.03217		7.821	7.6381	8.00458		7.868	7.7108	8.02597		0.024	–0.1619	0.20988	
4.096	3.9931	4.19972		6.691	6.5495	6.83174		7.609	7.4421	7.7764		7.687	7.5436	7.83118		0.024	–0.1397	0.18778	
RAM and 95% CI			*P*-value	RAM and 95% CI			*P*-value	RAM and 95% CI			*P*-value	RAM and 95% CI			*P*-value	RAM and 95% CI			*P*-value
4.088	3.9632	4.21223	0.219	6.869	6.6943	7.04415	0.228	7.749	7.5435	7.95503	0.649	7.788	7.612	7.96493	0.839	0.097	–0.0982	0.29246	0.374
4.199	4.0974	4.3015		6.713	6.5717	6.85514		7.691	7.5248	7.85811		7.785	7.6418	7.92776		0.059	–0.1082	0.22708	
4.05	3.9451	4.15427		6.663	6.5167	6.8097		7.61	7.4372	7.78275		7.717	7.57	7.86498		–0.019	–0.1836	0.14627	
4.139	4.019	4.25928		6.638	6.4728	6.803		7.594	7.3991	7.7882		7.709	7.5417	7.87545		–0.121	–0.3076	0.06571	
RAM and 95% CI			*P*-value	RAM and 95% CI			*P*-value	RAM and 95% CI			*P*-value	RAM and 95% CI			*P*-value	RAM and 95% CI			*P*-value
4.108	4.0286	4.18727	0.895	6.712	6.601	6.82289	0.810	7.657	7.5268	7.78741	0.737	7.722	7.611	7.83369	0.722	0.045	–0.0838	0.17472	0.191
4.135	4.0419	4.2283		6.737	6.6073	6.86642		7.628	7.4772	7.77973		7.762	7.6325	7.8917		–0.094	–0.236	0.04839	
4.134	3.9841	4.28451		6.657	6.45	6.86339		7.742	7.4972	7.98748		7.816	7.6058	8.02596		0.124	–0.1005	0.34799	

*Significant.

**Significant with Bonferroni correction.

*RAM* Regression-adjusted mean, *CI* Confidence interval, *HS* High school, *DHEA* Dehydroepiandrosterone, *AEA N*-arachidonoylethanolamine, *1-Ag* 1-Arachidonoylglycerol, *SEA* stearoylethanolamine, *OEA* oleoylethanolamide, *PEA* palmitoylethanolamide, *PedBE* Pediatric-Buccal-Epigenetic Clock.

**Table 3:** Logistic Regressions Predicting Being in the High Inflammatory Cluster by Social Adversity

Social Variable, N = 449	OR (95% CI)	*P*-value
Education		
<High School	1.27 (0.62–2.57)	0.513
Some HS	0.79 (0.36–1.71)	0.544
High School	0.83 (0.40–1.72)	0.610
>High School	Ref	–
Income		
$0–$5K	0.91 (0.43–1.92)	0.809
$5001–$15K	0.82 (0.39–1.69)	0.583
$15,001-$25K	1.52 (0.77–3.00)	0.222
$25,001 or more	Ref	–
Social Risks		
0–1	1.51 (0.73–3.10)	0.263
2–3	0.54 (0.24–1.19)	0.124
4–6	0.82 (0.39–1.70)	0.592
7 or more	Ref	–
Cumulative Social Adversity		
2–3	2.27 (1.18–4.38)	0.014*
1	0.83 (0.46–1.51)	0.539
0	Ref	–

*Significant.

*OR* Odds ratio, *CI* Confidence interval.

There were few associations between biomarkers and either household income or social risks. One exception was SEA, which showed a U-shaped association: those from the middle two income groups had higher SEA values than those with the highest or lowest income (*P* = 0.013). The other exception involved IL-1β, which was significantly associated with social risks (*P* = 0.028). Households experiencing either the fewest or most social risks had higher values of IL-1β compared to those with the middle two categories of social risks (Table 2).

### Associations between biomarkers and cumulative social adversity

Cumulative social adversity was significantly associated with IL-1β (*P* < 0.001), IL-6 (*P* = 0.001), IL-8 (*P* = 0.003), TNF-α (*P* = 0.006), and cortisol (*P* = 0.045). For all inflammatory markers, those with a cumulative social adversity score of 0 had lower levels of inflammatory markers than those with scores of 2 or 3. Those with a cumulative social adversity score of 1 had a higher average level of cortisol than those with a score of 0 or 2-3. There were no significant relationships between cumulative social adversity and cortisone, endocannabinoids, nor PedBE clock (Table 2). Cumulative social adversity was also significantly associated with being in the high inflammatory cluster. Those with a cumulative social adversity score of 2-3 had greater odds of being in the high inflammatory cluster compared to those with a score of 0 (odds ratio 2.27, 95% confidence interval 1.18–4.38, *P* = 0.014).

### Sensitivity analyses

Results from sensitivity analyses adjusting for inflammatory conditions were largely unchanged (Tables 4 and 5). In sensitivity analyses adjusting for race and ethnicity the relationship between caregiver education and child OEA (*P* = 0.218) and between child cortisol and cumulative adversity (*P* = 0.123) became not statistically significant (Supplementary Table 2). Otherwise, results did not change substantially when adjusting for race and ethnicity (Supplementary Tables 2 and  3).

**Table 4:** Relationships between Social Adversity and Child Biomarkers, Adjusting for Inflammatory Conditions

	IL-1β				IL-6				IL-8				TNF-alpha			
Social Variable	*N* = 484				*N* = 479				*N* = 484				*N* = 480			
**Education**	**RAM and 95% CI**			***P*-value**	**RAM and 95% CI**			***P*-value**	**RAM and 95% CI**			***P*-value**	**RAM and 95% CI**			***P*-value**
<High School	3.67182	3.4707	3.87294	0.044*	1.316	1.11526	1.51673	0.015*	6.166	5.98905	6.34295	0.168	0.30103	0.15167	0.45039	0.202
Some HS	3.44837	3.23966	3.65709		1.05374	0.84703	1.26044		6.05833	5.87469	6.24196		0.3012	0.14546	0.45694	
High School	3.32449	3.13245	3.51654		0.9172	0.72719	1.10722		5.97317	5.8042	6.14214		0.24953	0.10585	0.3932	
>High School	3.30076	3.08683	3.51469		0.89942	0.68854	1.11031		5.88518	5.69695	6.0734		0.09075	–0.06887	0.25037	
**Income**	**RAM and 95% CI**			***P*-value**	**RAM and 95% CI**			***P*-value**	**RAM and 95% CI**			***P*-value**	**RAM and 95% CI**			***P*-value**
$0–$5K	3.48064	3.25963	3.70165	0.398	1.10787	0.88828	1.32745	0.602	6.08939	5.89549	6.28328	0.449	0.25062	0.08545	0.41578	0.195
$5001–$15K	3.47425	3.2749	3.6736		0.9663	0.7683	1.1643		6.03734	5.86245	6.21223		0.24752	0.10028	0.39475	
$15,001–$25K	3.52385	3.31116	3.73654		1.13484	0.92342	1.34625		6.08244	5.89584	6.26904		0.35826	0.20045	0.51606	
$25,001 or more	3.29883	3.10811	3.48955		0.9987	0.8085	1.18889		5.90994	5.74262	6.07726		0.12561	–0.01581	0.26702	
**Social Risks**	**RAM and 95% CI**			***P*-value**	**RAM and 95% CI**			***P*-value**	**RAM and 95% CI**			***P*-value**	**RAM and 95% CI**			***P*-value**
0–1	3.53648	3.30836	3.76461	0.028*	0.97848	0.75038	1.20657	0.395	6.01504	5.81375	6.21633	0.346	0.22016	0.04967	0.39066	0.227
2–3	3.22919	3.04249	3.4159		1.06424	0.8772	1.25128		5.93343	5.76869	6.09818		0.17447	0.03461	0.31433	
4–6	3.42278	3.23364	3.61192		0.95861	0.76897	1.14825		6.0108	5.8439	6.17769		0.20548	0.06482	0.34613	
7 or more	3.64776	3.42927	3.86624		1.19463	0.9773	1.41196		6.16638	5.9736	6.35916		0.38902	0.22578	0.55226	
**Cumulative Social Adversity**	**RAM and 95% CI**			***P*-value**	**RAM and 95% CI**			***P*-value**	**RAM and 95% CI**			***P*-value**	**RAM and 95% CI**			***P*-value**
0	3.33878	3.19231	3.48525	0.001**	0.95739	0.81183	1.10294	0.001*	5.96497	5.83614	6.0938	0.003*	0.22655	0.11746	0.33565	0.006*
1	3.37587	3.21147	3.54026		0.97933	0.8158	1.14287		5.94846	5.80387	6.09306		0.14357	0.02022	0.26692	
2–3	3.91216	3.6492	4.17512		1.50298	1.24107	1.76489		6.39977	6.16848	6.63105		0.52047	0.32501	0.71592	

Cortisol				Cortisone				Progesterone				DHEA				AEA			
*N* = 406				*N* = 409				*N* = 245				*N* = 320				*N* = 400			
**RAM and 95% CI**			***P*-value**	**RAM and 95% CI**			***P*-value**	**RAM and 95% CI**			***P*-value**	**RAM and 95% CI**			***P*-value**	**RAM and 95% CI**			***P*-value**
3.50798	3.15038	3.86558	0.419	3.32452	3.17993	3.46912	0.191	0.69145	0.62668	0.75622	0.364	1.97689	1.71076	2.24302	0.304	–0.12057	–0.20071	–0.0404	0.001**
3.92417	3.56414	4.2842		3.50669	3.36083	3.65255		0.68538	0.62671	0.74405		1.89095	1.64266	2.13924		0.10808	0.02729	0.18888	
3.6867	3.34581	4.02758		3.30298	3.16743	3.43853		0.63112	0.56881	0.69342		2.0454	1.80371	2.28709		-0.06711	–0.1435	0.00928	
3.61239	3.23626	3.98853		3.36039	3.21048	3.5103		0.70716	0.6414	0.77292		1.70429	1.42859	1.97999		–0.05034	–0.13567	0.035	
RAM and 95% CI			*P*-value	RAM and 95% CI			*P*-value	RAM and 95% CI			*P*-value	RAM and 95% CI			*P*-value	RAM and 95% CI			*P*-Value
3.62664	3.21363	4.03964	0.978	3.32161	3.15754	3.48567	0.398	0.66942	0.59985	0.73898	0.983	2.14369	1.85165	2.43574	0.154	0.05788	–0.03519	0.15094	0.071
3.72549	3.3726	4.07838		3.34891	3.2081	3.48971		0.67345	0.61563	0.73127		2.01664	1.76561	2.26766		-0.00731	–0.08699	0.07238	
3.71919	3.35979	4.07859		3.31594	3.1698	3.46208		0.68357	0.61642	0.75072		1.80427	1.54759	2.06096		-0.06018	–0.14274	0.02238	
3.6535	3.32569	3.98132		3.46509	3.33359	3.59658		0.68516	0.62541	0.74491		1.7659	1.52974	2.00206		-0.09323	–0.16772	-0.0187	
RAM and 95% CI			*P*-value	RAM and 95% CI			*P*-value	RAM and 95% CI			*P*-value	RAM and 95% CI			*P*-value	RAM and 95% CI			*P*-Value
3.58575	3.18334	3.98816	0.425	3.39093	3.22968	3.55219	0.230	0.71653	0.6483	0.78477	0.162	1.75597	1.45831	2.05362	0.517	–0.0671	–0.15781	0.0236	0.220
3.72407	3.40007	4.04806		3.43913	3.30693	3.57133		0.68503	0.62611	0.74395		1.95344	1.72009	2.18679		–0.02649	–0.10115	0.04817	
3.87712	3.53815	4.21608		3.39202	3.25731	3.52673		0.62464	0.5666	0.68268		2.02364	1.78669	2.26058		-0.0739	–0.15079	0.00299	
3.46788	3.08763	3.84812		3.23467	3.08301	3.38632		0.70291	0.63661	0.7692		1.84525	1.56883	2.12167		0.04072	–0.04689	0.12834	
RAM and 95% CI			*P*-value	RAM and 95% CI			*P*-value	RAM and 95% CI			*P*-value	RAM and 95% CI			*P*-value	RAM and 95% CI			*P*-Value
3.73082	3.47832	3.98332	0.043*	3.41136	3.30925	3.51346	0.063	0.68208	0.63768	0.72648	0.127	1.84185	1.6598	2.0239	0.480	-0.0495	–0.10787	0.00887	0.665
3.82987	3.53453	4.1252		3.3961	3.27796	3.51424		0.64612	0.59424	0.69801		2.0127	1.80211	2.22329		-0.02674	–0.09453	0.04105	
3.13371	2.65999	3.60744		3.15808	2.96658	3.34958		0.74592	0.6636	0.82824		1.91123	1.54894	2.27351		0.00528	–0.10402	0.11458	

1-Ag				SEA				OEA				PEA				PedBE			
*N* = 403				*N* = 401				*N* = 410				*N* = 411				*N* = 449			
**RAM and 95% CI**			***P*-value**	**RAM and 95% CI**			***P*-value**	**RAM and 95% CI**			***P*-value**	**RAM and 95% CI**			***P*-value**	**RAM and 95% CI**			***P*-value**
4.10926	3.99731	4.22121	0.302	6.87699	6.72292	7.03106	0.082	7.86106	7.67839	8.04373	0.040*	7.90321	7.74649	8.05993	0.102	0.06779	–0.10514	0.24072	0.001*
4.20528	4.09201	4.31856		6.7017	6.54437	6.85902		7.69852	7.51448	7.88256		7.72685	7.56981	7.88388		–0.29285	–0.46546	–0.1202	
4.11634	4.00971	4.22297		6.66832	6.52086	6.81578		7.57019	7.39833	7.74204		7.73859	7.59113	7.88605		0.07064	–0.0991	0.24038	
4.04901	3.93242	4.1656		6.59552	6.43257	6.75847		7.50561	7.31449	7.69673		7.61906	7.45507	7.78305		0.18668	0.00534	0.36802	
RAM and 95% CI			*P*-value	RAM and 95% CI			*P*-value	RAM and 95% CI			*P*-value	RAM and 95% CI			*P*-value	RAM and 95% CI			*P*-value
4.13304	4.00424	4.26185	0.929	6.48696	6.30929	6.66464	0.014*	7.47797	7.26991	7.68602	0.098	7.68615	7.50743	7.86488	0.351	0.06989	–0.11518	0.25496	0.595
4.14535	4.03618	4.25452		6.73822	6.58668	6.88975		7.69064	7.51191	7.86938		7.74913	7.59634	7.90191		–0.09896	–0.27611	0.07819	
4.11122	3.99815	4.2243		6.87599	6.72175	7.03022		7.8208	7.63738	8.00422		7.86713	7.70957	8.02469		0.02094	–0.16499	0.20688	
4.09779	3.99445	4.20112		6.69389	6.55268	6.83509		7.61049	7.44306	7.77792		7.69009	7.54626	7.83391		0.02767	–0.1362	0.19154	
RAM and 95% CI			*P*-value	RAM and 95% CI			*P*-value	RAM and 95% CI			*P*-value	RAM and 95% CI			*P*-value	RAM and 95% CI			*P*-Value
4.0855	3.96095	4.21005	0.224	6.86663	6.69172	7.04154	0.237	7.74764	7.5416	7.95368	0.658	7.78515	7.60864	7.96165	0.852	0.09536	–0.10006	0.29077	0.386
4.19775	4.09568	4.29983		6.71136	6.56966	6.85307		7.69063	7.52377	7.8575		7.78318	7.64023	7.92613		0.05953	–0.10812	0.22718	
4.04965	3.94508	4.15422		6.6639	6.51745	6.81034		7.61034	7.4374	7.78329		7.71772	7.57027	7.86518		–0.01853	–0.18352	0.14645	
4.13909	4.01899	4.2592		6.63745	6.47244	6.80245		7.59355	7.39882	7.78828		7.70832	7.5415	7.87513		–0.11926	–0.30599	0.06747	
RAM and 95% CI			*P*-value	RAM and 95% CI			*P*-value	RAM and 95% CI			*P*-value	RAM and 95% CI			*P*-value	RAM and 95% CI			*P*-Value
4.10634	4.02699	4.18568	0.885	6.71027	6.59937	6.82116	0.812	7.65609	7.52562	7.78656	0.737	7.72014	7.60878	7.8315	0.708	0.04339	–0.08596	0.17275	0.195
4.13434	4.04115	4.22753		6.73621	6.60671	6.8657		7.62837	7.47695	7.77979		7.76188	7.63231	7.89144		–0.09234	–0.23459	0.04992	
4.13488	3.98472	4.28504		6.6568	6.45022	6.86337		7.74241	7.49705	7.98777		7.81599	7.60604	8.02594		0.12682	-0.09757	0.35121	

*Significant.

**Significant with Bonferroni correction.

*RAM* Regression-adjusted mean, *CI* Confidence interval, *HS* High school, *DHEA* Dehydroepiandrosterone, *AEA* N-arachidonoylethanolamine, *1-Ag* 1-Arachidonoylglycerol, *SEA* stearoylethanolamine, *OEA* oleoylethanolamide, *PEA* palmitoylethanolamide, *PedBE* Pediatric-Buccal Epigenetic Clock.

**Table 5:** Logistic Regressions Predicting Being in the High Inflammatory Cluster by Social Adversity, Adjusting for Inflammatory Conditions.

Social Variable, *N* = 449	OR (95% CI)	*P*-value
Education		
<High School	1.27 (0.62–2.57)	0.513
Some HS	0.79 (0.36–1.71)	0.545
High School	0.83 (0.40–1.72)	0.610
>High School	Ref	–
Income		
$0–$5K	0.91 (0.43–1.92)	0.807
$5001–$15K	0.82 (0.39–1.69)	0.586
$15,001–$25K	1.53 (0.39–3.0)	0.221
$25,001 or more	Ref	–
Social Risks		
0–1	1.51 (0.73–3.10)	0.263
2–3	0.54 (0.24–1.19)	0.124
4–6	0.82 (0.39–1.70)	0.593
7 or more	Ref	–
Cumulative Social Adversity		
2–3	2.27 (1.18-4.38)	0.014*
1	0.83 (0.46–1.51)	0.540
0	Ref	–

*Significant.

*OR* Odds ratio, *CI* Confidence interval.

## Discussion

We found statistically significant associations between cumulative social adversity and inflammation in children as measured by multiple cytokines. Children experiencing more social adversity had higher levels of several inflammatory markers. We found few significant associations with specific types of social adversity (only parental education). Our findings strengthen prior work showing that adversity is related to elevated inflammatory markers among children and, moreover, that more adversity is related to increased odds of worse health.^2,3^ They also support the rationale for clinical activities focused on identifying social adversity and addressing social needs.^32^ To our knowledge, this is one of the few studies to demonstrate associations between cumulative social adversity and cytokine measures, and the only study showing relationships between social adversity and cytokine clusters. Our findings are in line with two systematic reviews and meta-analyses on associations between early adversity and inflammatory markers in children and adolescents that described associations between childhood adversity and inflammation as measured by elevated C-reactive protein (CRP) and IL-6, although in the review the association with IL-6 did not reach significance.^13,14^ One of the studies concluded that evidence supporting these associations is limited by the paucity of studies and the heterogeneity of methods.^14^

We also found that child cortisol was associated with cumulative social adversity: children with a cumulative social adversity tally of 1 had a higher average log cortisol than those with a cumulative adversity tally of 0 or 2-3 social risk factors. Those with a tally of 0 likely have lower levels of cortisol than those with 1 due to less exposure to social adversity. Prior work has shown that levels of cortisol are different in children experiencing social adversity—sometimes higher and sometimes lower—than in children not experiencing social adversity.^17^ This finding has been attributed to differences in acute versus chronic stress exposure, with those experiencing acute stressors having higher levels of cortisol or cortisone.^18^ Those with multiple social risk factors may experience blunting effects, whereby the capacity of the hypothalamic-pituitary-adrenal (HPA) axis to reduce to acute stressors is impaired, leading to decreased cortisol production, and subsequently, *lower* levels of cortisol than those with fewer social risk factors.^19^ Our finding that children with 2 or more cumulative social adversities had lower cortisol levels than those with a cumulative social adversity tally of 1 aligns with the possibility of those experiencing chronic stress experiencing blunting effects of the HPA axis. Future studies should assess both social adversity and cortisol at multiple timepoints to clarify the pathway from cumulative social stress to disordered biological stress response.

In contrast to several prior studies that have shown either elevated cortisone or decreased cortisone in the setting of stress,^33–35^ we found no significant relationships between specific types of social adversities and cortisone, although we did find a borderline significant association between cumulative social adversity and cortisone (*P* = 0.062): cortisone decreased as cumulative social adversity increased. This finding is consistent with theories about the HPA axis blunting effects on cortisol and cortisone production in the setting of chronic stress. As an example, Muenter et al. (2021) found that children who witnessed their fathers being arrested were more likely to have acutely high cortisol and cortisone levels, but that children with ongoing behavioral stress symptoms (a proxy for more chronic stress exposure) had lower cortisol and cortisone levels.^35^

Besides cortisol and cortisone, other steroids were not consistently associated with either specific or cumulative social adversity. Our null DHEA findings contrast with prior work showing that prolonged stress is associated with lower levels of DHEA in adults.^36^ Our null progesterone findings support some prior work among children, and are in contrast to work conducted among adults.^22,24^ In summary, there have been mixed findings about the relationship between steroids and social adversity. Our study contributes one of the largest studies of the relationship between stress and steroids among children of which we are aware, although larger and more diverse samples are needed to understand the complex relationship between steroids and both acute and chronic stress exposure.

We did not find consistent associations between endocannabinoids and either specific or cumulative social adversity, suggesting that social adversity may not influence the stress response via these neurotransmitters as strongly as through other mechanisms. This is contrary to our hypothesis, which we developed based on prior work demonstrating that in an animal model, mice experiencing early adversity generally had lower levels of endocannabinoids compared to those not experiencing adversity.^37^ It is worth noting that a small study of unaccompanied refugee minors showed inconsistent associations between endocannabinoids and stress exposure, which, paired with our results, may indicate that endocannabinoids may not be the most sensitive biomarker of childhood adversity.^38^ While this is one of the only studies of which we are aware to study associations between social adversity and endocannabinoid levels among children, more extensive work is needed to determine whether endocannabinoids are sensitive to social adversity exposure.

We also did not find a consistent association between PedBE clock and select or cumulative social adversity, suggesting that in children, social stress may not be reflected by changes in this biomarker. This is one of the few studies to assess the relationship between PedBE clock and social adversity among children. Our lack of significant associations related to PedBE may be in part due to the broad age range of our sample. While we did adjust for age in our analyses, it is possible that the PedBE clock operates differently based on different age ranges. Our findings may also be partly due to homogeneity of the sample, as participants were recruited from a single urgent care clinic that only serves patients who are publicly insured or uninsured (often proxies for low-income). It is possible in a more socially heterogenous sample we might have found significant associations between social risk factors and a range of biomarkers. Alternatively, PedBE clock age may not be as sensitive an epigenetic marker of social adversity as alternatives that measure the *pace* of aging, such as the DunedinPoAm approach. For instance, Raffington et al. (2021) found that while social adversity (in that study measured by neighborhood-level factors) was not related to DNA methylation age, it was associated with faster pace of aging.^39^

Our findings that inflammatory markers and certain steroids are associated with cumulative social adversity supports enthusiasm from groups like the American Academy of Pediatrics around using noninvasively collected biomarkers to inform clinical care.^40^ However, healthcare systems are not consistently using biomarkers to inform treatment and management decisions. But as the science improves, the usefulness of biomarkers is likely to increase. For instance, a recent spate of healthcare initiatives has focused on social risk screening and referrals to social services in the context of clinical encounters; some have argued that there is limited evidence on the health benefits of these activities.^41^ Our work indicates that these types of social risk screening and intervention protocols might especially benefit children with diseases exacerbated by inflammation and could potentially be measured using relevant biomarkers. For example, a recent study of children with childhood arthritis showed an association between cumulative social adversity and severity of disease, and is consistent with our data showing positive associations between cumulative social risk and inflammatory markers.^42^ Our work also suggests that when biomarker results can inform clinical care, it may be helpful to collect samples from children at highest risk of having results at the extremes, such as those experiencing chronic and cumulative social adversity. Just as policies have been implemented to encourage screening for social adversity through reimbursements or requirements, as biomarkers become more clinically meaningful to informing care, policies incentivizing stress biomarker screening among those most at risk for extreme values could encourage clinical practice change.^41,43–46^

Clinical teams’ awareness of patients’ social circumstances also can facilitate changes to medical care that reduce barriers to care.^47^ As the biological pathways between social adversity and specific health outcomes are elucidated, more information will emerge about effective social risk care adjustments. As just one example, we see socioeconomic disparities in sepsis outcomes. Evidence suggests avoiding steroid administration^48^ unless patients are on chronic steroids or have a corticosteroid-related endocrinopathy. But if chronic stress-related exposure to internally produced steroids like cortisol similarly affects the adrenal gland’s response to sepsis, children experiencing chronic stress may benefit from steroids in certain cases (although this would need to be studied carefully before trialing it). To clarify relationships between social stress, biomarkers, and clinical outcomes, future studies involving cytokine and steroid responses to disease should measure both individual types and cumulative levels of social adversity and how they may affect different disease processes.

### Limitations

Our study findings should be interpreted in light of several key limitations. First, the research is based on data from a cohort of patients from a single urban safety net clinic. It is possible that with a more socioeconomically diverse sample, we would have seen stronger associations between social adversity and biomarkers of disease. Second, our measures of social adversity and biomarkers were collected cross sectionally, which means we can only look at associations between these variables. We are not able to assess how changes in social adversity or chronic stress exposure may causally influence biomarker outcomes. Understanding timing and chronicity of adversity exposure will be important to future work, as O’Connor et al.^49^ found differences in the association between adversity and CRP when measured at different timepoints in childhood.^49^ Additionally, while our study included the measurement of many biomarkers, there are many others which we did not measure that could shed light on the relationship between adversity exposure and health outcomes among children. For example, prior work suggests that cumulative social adversity in childhood is associated with levels of glycoprotein acetyls, biomarkers considered to reflect cumulative inflammation.^49^ Also, while we did collect information on many social factors, similar to our biomarker collection, our social factor information was not exhaustive. There are other social factors such as caregiver partner/marital status and adverse childhood experiences that may show relationships with the biomarkers we measured and should be considered for collection in future work. Finally, although our study represents one of the larger studies of the association between social adversity and biomarkers, we are still limited by our sample size, especially given the age range of our sample.

### Conclusion

In a cohort of over 400 children, we found that higher cumulative social adversity was associated with increased levels of inflammation and decreased levels of cortisol. Our findings support prior work suggesting that social stress exposures contribute to increased inflammation in children. Future work should closely explore the multiple pathways between social stress, the physiologic response, and health outcomes.

### Supplementary information

Supplementary Table 1

Supplementary Table 2

Supplementary Table 3

## Data Availability

This paper uses data from a previous randomized clinical trial (RCT). After publication, deidentified participant data is available by request to researchers whose proposed use of the data has been approved. Requests can be made to holly.wing@ucsf.edu, and approval is at the discretion of the primary RCT research team, with signed data use agreement.
