# Supplementary material for: Associations between Social Adversity and Biomarkers of Inflammation, Stress, and Aging in Children
Source: Pediatr Res. 2024 Jan 17;95(6):1553–63. doi: 10.1038/s41390-023-02992-6 (PMC11126389; doi:10.1038/s41390-023-02992-6)
Supplement: Supplementary file 3 — Supplementary Table 3 [file 41390_2023_2992_MOESM3_ESM.pdf]

SUPPLEMENTAL TABLE 3. Logistic Regressions Predicting in the High Inflammatory Cluster by Social Adversity, Adjusting for Race and Ethnicity

Social Variable, N=449	OR (95% CI)	*P*-Value
Education		
< High School	1.28 (.60-2.73)	0.532
Some HS	.86 (.38-1.92)	0.705
High School	.84 (.39-1.80)	0.659
> High School	Ref	-
Income		
$0-$5K	.93 (.44-1.97)	0.847
$5,001-$15K	.84 (.40-1.76)	0.649
$15,001-$25K	1.60 (.80-3.18)	0.180
$25,001 or more	Ref	-
Social Needs		
0-1	1.40 (.67-2.92)	0.364
2-3	.50 (.22-1.12)	0.090
4-6	.82 (.39-1.71)	0.596
7 or more	Ref	-
Cumulative		
2-3	2.17 (1.11-4.26)	0.023*
1	.81 (.44-1.49)	0.506
0	Ref	-

* Significant

** Significant with Bonferroni correction
